# Quantifying the impact of key factors on the carbon mitigation potential of managed temperate forests

**DOI:** 10.1186/s13021-023-00247-9

**Published:** 2024-03-02

**Authors:** Konstantin Gregor, Andreas Krause, Christopher P. O. Reyer, Thomas Knoke, Benjamin F. Meyer, Susanne Suvanto, Anja Rammig

**Affiliations:** 1 TUM School of Life Sciences, Technical University of Munich, Freising, Germany; 2 Potsdam Institute for Climate Impact Research, Member of the Leibniz Association, Potsdam, Germany; 3 Natural Resources Institute Finland (Luke), Helsinki, Finland; 4 School of Geography, Earth and Environmental Sciences, University of Birmingham, Birmingham, United Kingdom; 5 Birmingham Institute of Forest Research, University of Birmingham, Birmingham, United Kingdom

**Keywords:** Climate change, Carbon mitigation, Forest, Substitution effect, Displacement factor, Decarbonization, Disturbance, Salvage logging, Wood usage

## Abstract

**Background:**

Forests mitigate climate change by reducing atmospheric $\mathrm {CO_2}$-concentrations through the carbon sink in the forest and in wood products, and substitution effects when wood products replace carbon-intensive materials and fuels. Quantifying the carbon mitigation potential of forests is highly challenging due to the influence of multiple important factors such as forest age and type, climate change and associated natural disturbances, harvest intensities, wood usage patterns, salvage logging practices, and the carbon-intensity of substituted products. Here, we developed a framework to quantify the impact of these factors through factorial simulation experiments with an ecosystem model at the example of central European (Bavarian) forests.

**Results:**

Our simulations showed higher mitigation potentials of young forests compared to mature forests, and similar ones in broad-leaved and needle-leaved forests. Long-lived wood products significantly contributed to mitigation, particularly in needle-leaved forests due to their wood product portfolio, and increased material usage of wood showed considerable climate benefits. Consequently, the ongoing conversion of needle-leaved to more broad-leaved forests should be accompanied by the promotion of long-lived products from broad-leaved species to maintain the product sink. Climate change (especially increasing disturbances) and decarbonization were among the most critical factors influencing mitigation potentials and introduced substantial uncertainty. Nevertheless, until 2050 this uncertainty was narrow enough to derive robust findings. For instance, reducing harvest intensities enhanced the carbon sink in our simulations, but diminished substitution effects, leading to a decreased total mitigation potential until 2050. However, when considering longer time horizons (i.e. until 2100), substitution effects became low enough in our simulations due to expected decarbonization such that decreasing harvests often seemed the more favorable solution.

**Conclusion:**

Our results underscore the need to tailor mitigation strategies to the specific conditions of different forest sites. Furthermore, considering substitution effects, and thoroughly assessing the amount of avoided emissions by using wood products, is critical to determine mitigation potentials. While short-term recommendations are possible, we suggest risk diversification and methodologies like robust optimization to address increasing uncertainties from climate change and decarbonization paces past 2050. Finally, curbing emissions reduces the threat of climate change on forests, safeguarding their carbon sink and ecosystem services.

**Supplementary Information:**

The online version contains supplementary material available at 10.1186/s13021-023-00247-9.

## Background

Forests are pivotal in the fight against global climate change due to their significant role in the global carbon cycle [1, 2]. Most obviously, forests mitigate climate change through the in situ forest carbon sink which sequestered 2.4 $\text {PgC yr}^{-1}$ or roughly 22% of anthropogenic $\mathrm {CO_2}$-emissions in recent decades [3, 4]. However, in places like Europe, where about 75% of forests are used for wood production [5], at least two other aspects are highly relevant. One is the wood product carbon sink which in Europe accounts for about 13% of the forest carbon sink, the other is the substitution of carbon-intensive fuels and materials with wood products [6]. In Europe, this currently had a similar magnitude as the combined carbon sink [7], but see our remarks on this in sections *Model evaluation* and *Decarbonization*].

The exact magnitudes of the forest and wood product carbon sinks and the substitution effects, however, depend on a multitude of factors. The forest sink depends on the forest type and age, forest management, climate change, and natural disturbances. For example, the European forest sink has been shown to be in decline due to ageing and increased disturbances [8, 9]. The wood product sink depends on the same factors, but in addition, the product types and their lifetimes play a critical role. Moreover, because of more frequent disturbances, an increasing amount of timber from salvage logging affects wood quality and availability [10]. Finally, substitution effects depend on the carbon intensity of the replaced products. Though highly important, studies have suggested that these effects may have previously been overestimated, partly because the replaced materials and fuels will likely become less carbon-intensive in the future, e.g., due to a different energy mix [11, 12]. In construction, for example, wood can replace concrete and steel which recently accounted for roughly 14% of global $\mathrm {CO_2}$-emissions [13, 14]. But also other materials such as plastics or aluminum can be substituted with wood with a carbon benefit [15]. The decarbonization of these materials has already been initiated, for instance through increased efficiencies and recycling, but also through the adoption of existing technologies and investments in innovation [13, 14, 16]. The exact speed of this decarbonization, however, remains uncertain.

On the other hand, new technologies in the wood industry offer the possibility of enhanced wood use in the construction sector [17–19]. This calls for investigating the climate impact of such enhanced wood usage. Several studies have already found mitigation benefits of increased long-term usage of wood in various regions [20–23]. However, these studies did not yet consider in full detail to what degree the impact of forest structure, climatic change, disturbance regimes, and changes to the substitution dynamics might affect their results.

The complexity of determining the carbon mitigation potential of forests has resulted in a debate over the role of forests and wood products as a natural climate solution [24]. While some studies indicate a mitigation benefit of stable or increased harvest intensities [25, 26] other studies highlight the potential of decreased harvest intensities for increased carbon sequestration [9, 23, 27]. Verkerk et al. [9] also recently highlighted how different forest-based mitigation measures might conflict with each other.

To address this complexity, we used a model-based factorial experiment with a well-established process-based ecosystem model to set up a framework for quantifying the impact of all previously introduced factors: forest age, forest type, climate change, nitrogen (N) deposition, disturbances, harvest intensity and salvage logging, wood usage patterns, and changes in the carbon-intensity of substituted products. Using forests in the state of Bavaria in central Europe as an example, we quantified the impact of each factor independently, as well as their interactions and uncertainties. We then contextualized the findings of other mitigation studies and discussed how our results may be used as groundwork for developing mitigation strategies.

## Methods

### Description of the process-based ecosystem model LPJ-GUESS

For our simulations, we used the process-based ecosystem model LPJ-GUESS v4.1. The model is driven by environmental conditions (temperature, precipitation, short-wave radiation, atmospheric $\mathrm {CO_2}$, nitrogen deposition) and models a detailed forest structure via cohorts of different age classes. LPJ-GUESS simulates photosynthesis, allocation, growth, competition, nutrient limitation, establishment, and mortality of plant functional types [28, 29]. These are represented by parameters governing phenology, growth, drought and shade tolerance, bioclimatic limits for establishment and mortality, and others. For each forest location, a number of replicate patches (we used 100) are simulated. These depict random samples of forests at different stages after disturbances. Disturbances are modeled stochastically, killing the entire vegetation of a patch. These represent stand-replacing disturbances such as windthrows or insect infestations, the main disturbance agents in central Europe [30, 31]. The other mortality mechanisms such as growth efficiency mortality and age-related mortality kill fractions of the cohorts. Dead biomass is moved to various litter pools and the litter and soil carbon-nitrogen dynamics are simulated following the CENTURY model [29, 32].

LPJ-GUESS contains a forest management module that allows detailed representation of forestry, including thinnings and partial harvests, clearcuts, wood products and their decay, residue outtake, re-establishment and planting [33]. A full description of LPJ-GUESS can be found in Smith et al. [29].

### Modeling protocol

**Fig. 1:**
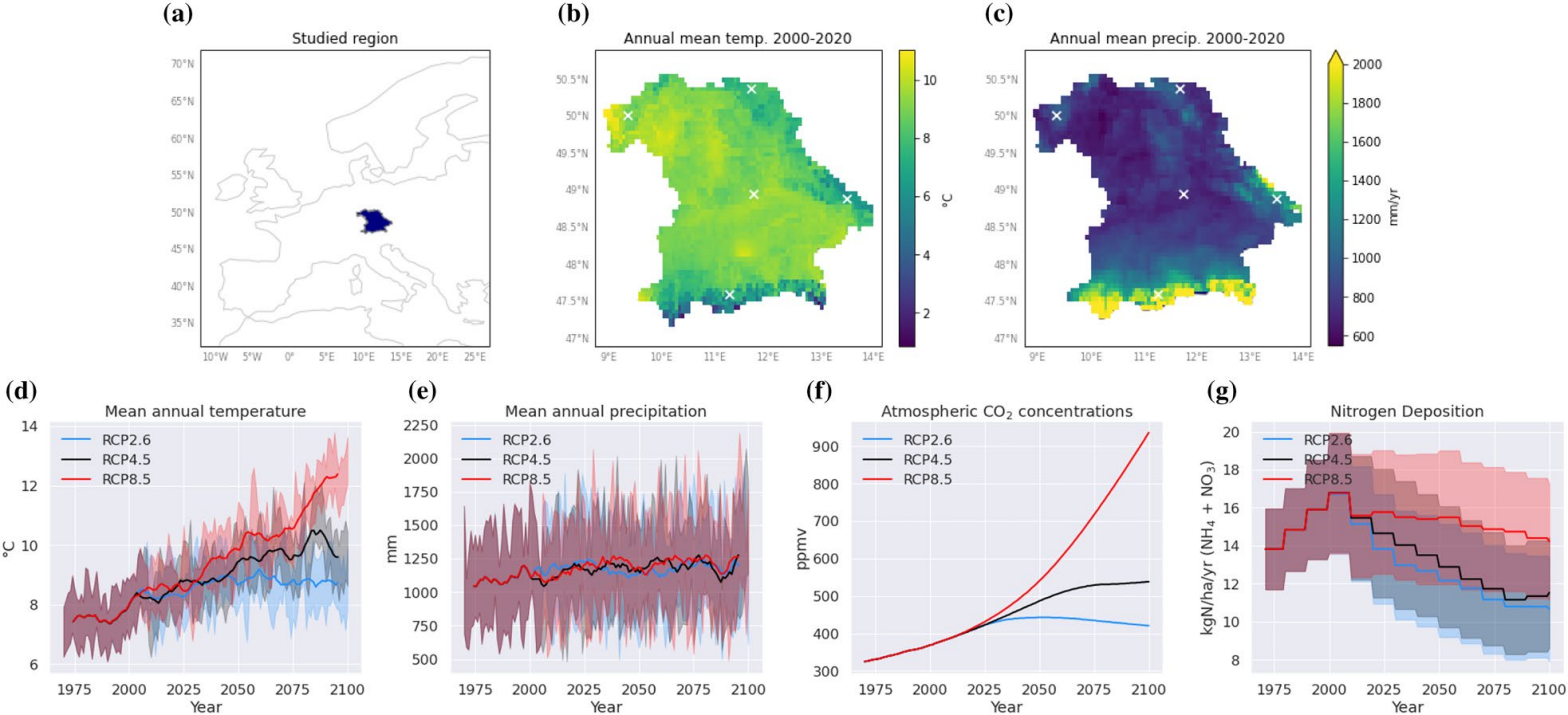
The study region is Bavaria, located in central Europe **a**, where we considered five sites (white crosses) for our simulation experiment. Mean annual temperature and precipitation are shown in panels (**b**) and (**c**). The forcing data **d**–**g** used as input for LPJ-GUESS shows 10 year rolling means averaged over the five sites with standard deviation bands for three emission scenarios (RCPs)

We conducted a simulation experiment for the federal state of Bavaria, Germany, in central Europe. We selected this region because of the detailed data availability on forest structure, harvests, wood usage, and product pools [e.g., 34]. For computational reasons, we selected five grid cells, covering the differences in regional climate (Fig. 1). All of these grid cells contained both needle-leaved evergreen (NE) and broad-leaved deciduous (BD) forests in 2018 according to the CORINE land cover data [35]. We used the plant functional types of shade-tolerant broad-leaved summergreen trees and shade-tolerant needle-leaved evergreen trees as a representation of the most dominant tree species in Bavaria. We used the default parameters as in [29] but used C/N ratios of sapwood and fine roots of 32 (53) and 661 (373) for the needle-leaved (broad-leaved) species, respectively [36, 37]. This was necessary to make LPJ-GUESS, which is calibrated mostly for global or continental applications, capture the high productivity of forests in central Europe (see section *Model evaluation*).

For the climate input, we used daily values of temperature, precipitation, and shortwave radiation from the regional climate model RACMO driven by the global climate model EC-EARTH [38, 39] from the EURO-CORDEX project [40]. The data were bias-corrected using quantile mapping and statistically down-scaled from 12.5 to 5 km resolution [41]. The climate data was available from 1951 to 2100. We initialized the model with a 1200 year spin-up period by recycling climate data from 1951–1980 to bring the carbon pools close to equilibrium. Yearly $\text{CO}_{{2}}$ concentrations and decadal values for nitrogen deposition were taken from [42].

We considered forest-type-specific disturbance intervals, i.e. we set the disturbance interval to 300 years for needle-leaved and 1000 years for broad-leaved forests [43]. Additionally, we assumed three different scenarios of disturbances in the future (see below). Fire was not explicitly simulated in this study since it is contained in the disturbance intervals and also not an important disturbance agent in the studied region [10, 30].

For our simulation experiment, we varied the main factors that govern the forest and product carbon sink and substitution effects: forest age, forest type, harvest intensity, salvage logging, wood usage patterns, climate change and nitrogen deposition, disturbances, and decarbonization of substituted products, as described in detail below. We simulated all 3456 possible combinations of the factors (Table 1).

**Table 1:** The considered values of the factors used in this study

Factor	Values	Comment
Climate change and N deposition	RCP2.6, RCP4.5, RCP8.5	See Fig. 1
Disturbance probability change ($*$)	Constant, linear, exponential	Changes in disturbance frequency based on temperature anomaly (Additional file 1: Fig. S1)
Forest age	Mature, young	Planted between 1921 and 1940, or between 1981 and 2000, respectively (Additional file 1: Fig. S2)
Forest type	BD, NE	Broad-leaved deciduous, needle-leaved evergreen forests
Harvest intensity	0%, 50%, 100%, 150%	Direct change in harvest intensity starting after 2020 compared to current values
Salvage logging	Yes, no	After every disturbance after 2020
Material wood usage	100%, 150%	The increase to 150% was implemented as a linear change from 2020 until 2050 at the expense of short-lived products and firewood
Cascade usage	100%, 150%	The change to 150% was implemented as a direct change of the lifetime of products created after 2020
Decarbonization in 2050	25%, 50%, 75%	Exponential decrease based on [44], reaching the given percentage value in 2050 (Additional file 1: Fig. S5)

All possible combinations were simulated, leading to $3 \times 3 \times 2 \times 2 \times 4 \times 2 \times 2 \times 2 \times 3 = 3456$ simulations

(*) Note that we used the exponential increase as the default in our analyses unless stated otherwise

### Considered factors for the carbon mitigation potential of forests

#### Climate change

To consider the full range of potential climate pathways and to make our assessment as broad as possible, we ran the simulations for the low-warming scenario RCP2.6, the intermediate scenario RCP4.5, and the high-warming scenario RCP8.5. This encompasses the projections of climate change according to current trends (2.1 to 3.9 °C global warming by 2100, 90% confidence interval [45]). Assessing wide ranges of climate change scenarios in preparation for low likelihood outcomes remains important, particularly given the remaining uncertainty of positive climate feedbacks [46, 47].

#### Disturbances

We implemented three scenarios of future changes to disturbance probabilities. Throughout this paper, we focused on an exponential increase in disturbance rates with temperature, based on recent observations of increased disturbances in Germany [10, Additional file 1: Fig. S1]. For an additional assessment, we included a linear increase and constant rates.

#### Forest age

We investigated the impact of “young” and “mature” forests. For this, forests were planted in each model run yearly over the 1981–2000 and the 1921–1940 period, respectively. The implemented harvesting (see below) allowed for the continuous establishment of young trees, resulting in structured, uneven-aged forest stands (Additional file 1: Fig. S2).

#### Forest type

In the plantings of forests in 1921–1940 and 1981–2000, only the broad-leaved or the needle-leaved plant functional type were planted to model BD and NE forests separately, depending on the model run (Table 1).

#### Harvest intensity

We simulated partial timber harvests to occur on average every 20 (NE) and 25 (BD) years with an intensity of 24% for all age cohorts and species, i.e. 24% of the trees of each age cohort were harvested. These are average numbers derived from recent German forest inventory data [48]. This setup resulted in stable growing stocks and describes a forward-looking forest management strategy [49, 50]. For the alternative scenarios, harvest intensity was then either decreased or increased by 50% starting directly after 2020, while the harvest intervals remained constant. When increasing the harvest intensity, stable growing stocks could no longer be guaranteed. We furthermore included a scenario without wood harvests after 2020.

During harvest events, 65% of the sapwood and heartwood of a selected tree were considered stem material of which 90% (“harvest efficiency”) were removed. For trees older than 20 years, the harvested stem wood was distributed to different product pools based on forestry statistics as described below. The rest of the stem wood, all coarse roots and 60% of the branches (13% of the woody biomass are considered branches) were assumed to be left to decay on-site. 40% of branches were assumed to be removed and burned [33, 51]. Trees younger than 20 years were completely used as firewood [52].

#### Salvage logging

We ran simulations both with and without salvage logging after a disturbance, taking into account the increased difficulty of harvest. For this, we assumed that 20% of the affected trees were left on site [53] and lowered the harvest efficiency, i.e., only 75% of a harvested stem was removed from the forest in a salvaging operation. We used the same usage patterns for salvaged wood as for fresh wood, because German wood use statistics indicate no substantial change in such patterns (Additional file 1: Fig. S3).

#### Wood products and substitution effects

For needle-leaved (broad-leaved) trees, 37% (6%) of harvested wood went into a long-lived product pool, 17% (34%) into a medium-lived pool, 36% (25%) into a short-lived pool; the rest was considered fuel wood and was returned to the atmosphere in the same year, following [54] and [26]. The decay of the products was modeled with Gamma-functions, such that 50% of the products have decayed after 3, 18, and 93 years for the short, medium, and long-lived pools, respectively [54]. To assess the effect of changes in material usage on the mitigation potential, we increased the fractions for medium- and long-lived products by 50% at the expense of short-lived products and firewood. This change in usage was implemented gradually from 2020 until 2050, based on current trends of construction wood usage in Germany [19]. To investigate the impact of more cascading (i.e., longer usage, more recycling), we also increased the residence time of all products by 50% by increasing the shape-parameter of the Gamma-functions (Additional file 1: Fig. S4) for products created after 2020.

For the substitution effects of wood products, we assumed a displacement factor (DF, avoided emissions in relation to the mass of carbon in the wood product [55]) of 1.5 tC/tC for material substitution and 0.67 tC/tC for fuel substitution [15]. Note that this DF $< 1$ means that if 1 tC of wood is harvested and used as fuel completely, the emission to the atmosphere is 1 tC and with a different energy source 0.67 tC would be emitted. The material DF does not contain end-of-life handling of the products. Since in Germany only 2% of waste is landfilled and wood is not allowed to be landfilled, we assumed full energy recovery of all products using again the DF for fuel.

#### Decarbonization

To assess the impacts of different decarbonization paces of the substituted materials and fuels [12, 56], we used three exponential functions to gradually decrease the DFs by 25%, 50%, and 75% of today’s values in 2050 (Additional file 1: Fig. S5). For instance, in the 75% scenario, the substitution factor of 1.5 tC/tC became 0.375 tC/tC in 2050, and approached zero around 2100, meaning that at that time, the product had no more substitution effect at all. We used this approach because the 25% and 75% scenarios closely match recent projections for the EU’s carbon intensity for current policies and net-zero targets, respectively [44].

It is important to note that throughout the text, we refer to the decarbonization of the *local* economy and viewed it as independent of the emissions of the rest of the world (defined here through the RCPs). For instance, RCP8.5 combined with 75% decarbonization can be viewed as an edge case where the considered region implements strong mitigation policies, but the rest of the world misses current global mitigation targets and increases their reliance on fossil fuels.

### Assessment of the carbon mitigation potential

In our study, we investigated the impact of the aforementioned factors on the forests’ carbon mitigation potential. For this, we computed the combined carbon sink, i.e., the change in carbon stored in the forest (live and dead biomass, and soil) and in products between 2020 and 2100. Furthermore, we computed the total carbon mitigation potential defined as the combined carbon sink plus the cumulative avoided emissions from substitution between 2020 and 2100. We averaged over the grid cells and focused on the mid- and long-term mitigation potential (years 2050 and 2100).

We based our main analysis on the case of an exponential increase in disturbance probability and then, to assess the impact of each factor on the mitigation potential, we computed pair-wise differences within the simulations. We repeated this experiment for the two other assumptions on disturbance frequencies. For instance, to quantify the impact of salvage logging, we subtracted the mitigation potential of each simulation where salvage logging was disabled from the mitigation potential of its “partner simulation” where salvage logging was enabled but all other settings were the same. For all factors with two possible values, this led to 576 comparisons (e.g., using the 1152 simulations with the exponential disturbance scenario, there were 576 simulations with salvage logging, 576 simulations without). For increased harvest intensity, it led to 288 comparisons between the 288 simulations with 150% harvest intensity and the 288 simulations with 100% harvest intensity. The 576 simulations with 0% and 50% harvest intensity were ignored in this particular assessment (but used to assess the implications of a decrease in harvest intensity). Accordingly, 384 comparisons were available for each of the three decarbonization paces. For increased material usage, we compared the simulations with both increased shares of long-lived products and increased cascading to those of the default values.

Our approach gives an insight into the potential impact of, e.g., a change in harvest intensity, while also considering the uncertainty stemming from all other factors, e.g., climate change. This helps to disentangle and compare all considered driving factors.

## Results

**Table 2:** Yearly mitigation values in gC/$\text {m}^2$/year from this study and the literature

Source	Bavaria 2020–2025	Germany 2014	Europe 2018	Europe 2016–2018	Bavaria 2003–2008
	This study	[19]	[7]	[6]	[34]
Forest sink	138 (Mature only: 104)	148	63	49	138 (Veg. carbon only)
Product sink	15 (16)	8	6	6	50
Fuel Substitution	27 (35)	92	25 (Includes paper)		
Material Substitution	32 (43)	77	38 (Including energy end-use)		
Total Substitution	59 (78)	169	63		130
Total Forest Mitigation	212 (198)	325	132		318

Literature values were converted from ${\text{MtCO}_{2e}}$ to gC per $\text {m}^2$ forest area. The values for [6] represent those of forest remaining forest. Note that the substitution values are theoretical values only: A substitution effect was attributed to the entire wood production. Such a value can sensibly only be used in comparison to a baseline scenario. Values that are not comparable to this study due to differences in accounting were excluded

### Model evaluation

The simulations with default settings (current harvest intensities, wood usage, DFs, and salvage logging practices) on average (over forest types, ages, and RCPs) and in relative terms are in the range of current literature estimates (Table 2). Simulated forest productivity also resembled independent estimates of Bavarian forests: For 2000–2015, gross and net primary productivity in our study were on average simulated as 1492 and $714 \,\text {gC}/\text {m}^2/\text {yr}$, respectively, close to satellite-based estimates of 1444 and $687 \,\text {gC}/\text {m}^2/\text {yr}$, respectively [57–59]. Literature estimates of the present-day product sink for European regions range around 5 to 12% of the forest sink (vegetation, soil, and litter), but higher values have also been estimated for Bavaria, where forests are heavily managed and dominated by conifers providing long-lived products (Table 2). Our simulations resulted in a product sink of 11% of the forest sink. We simulated the total *theoretical* substitution effects to be 28% of the total mitigation potential (theoretical because they are computed without comparison to a baseline, see discussion section Decarbonization). Literature values range between 41 and 52%. Key reasons for the spread are the assumed substitution factors but also the forest age structure, changes in forest area, and wood usage patterns of the considered regions. The main reason for our low magnitude of substitution effects is that we considered only young and mature forests, with young forests having very low substitution effects due to their low volumes. For mature forests only, substitution effects accounted for 39% of the total carbon mitigation. Nevertheless, our setup resulted in comparable mitigation dynamics as estimated by these studies which increases confidence in our calculations of the mitigation potential of central European forests. The wide range of estimates in the literature, however, also underscores the relevance of our study.

Due to continuous cuttings and regeneration, the age structure of the mature forests was already quite diverse in 2020 with mean tree ages of 72 (NE) and 84 years (BD). Young forests were still quite homogeneous in 2020 with a mean tree age of 28 years (Additional file 1: Fig. S2). The density of the BD forests was lower which was mostly driven by many small trees in the NE forests ($\le 20\text {cm}$).

### Impacts of different factors on the mitigation potential

**Fig. 2:**
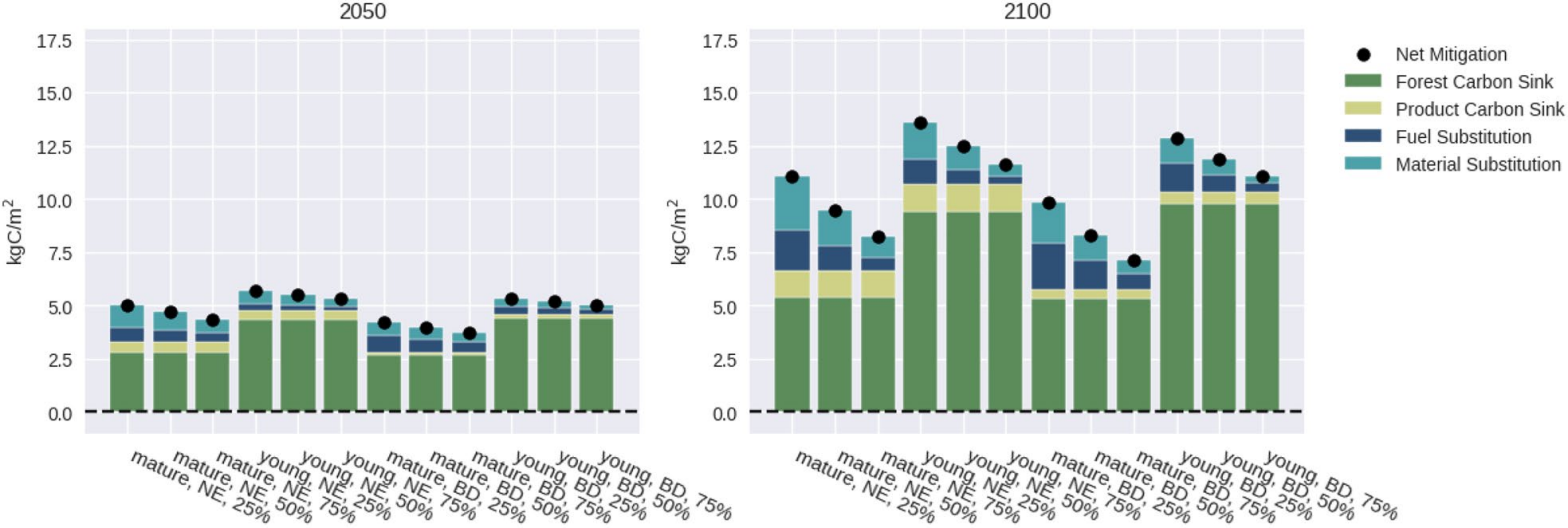
Contribution of each of the three aspects of mitigation to the total mitigation potential. Depicted is the situation for RCP4.5, without salvage logging and with harvest intensity, usage, and cascading at 100%. The different bars correspond to different combinations of forest age, forest type, and decarbonization pace. The dots represent total net mitigation and are the same values as the corresponding dots in Figs. 4 and Additional file 1: S8

**Fig. 3:**
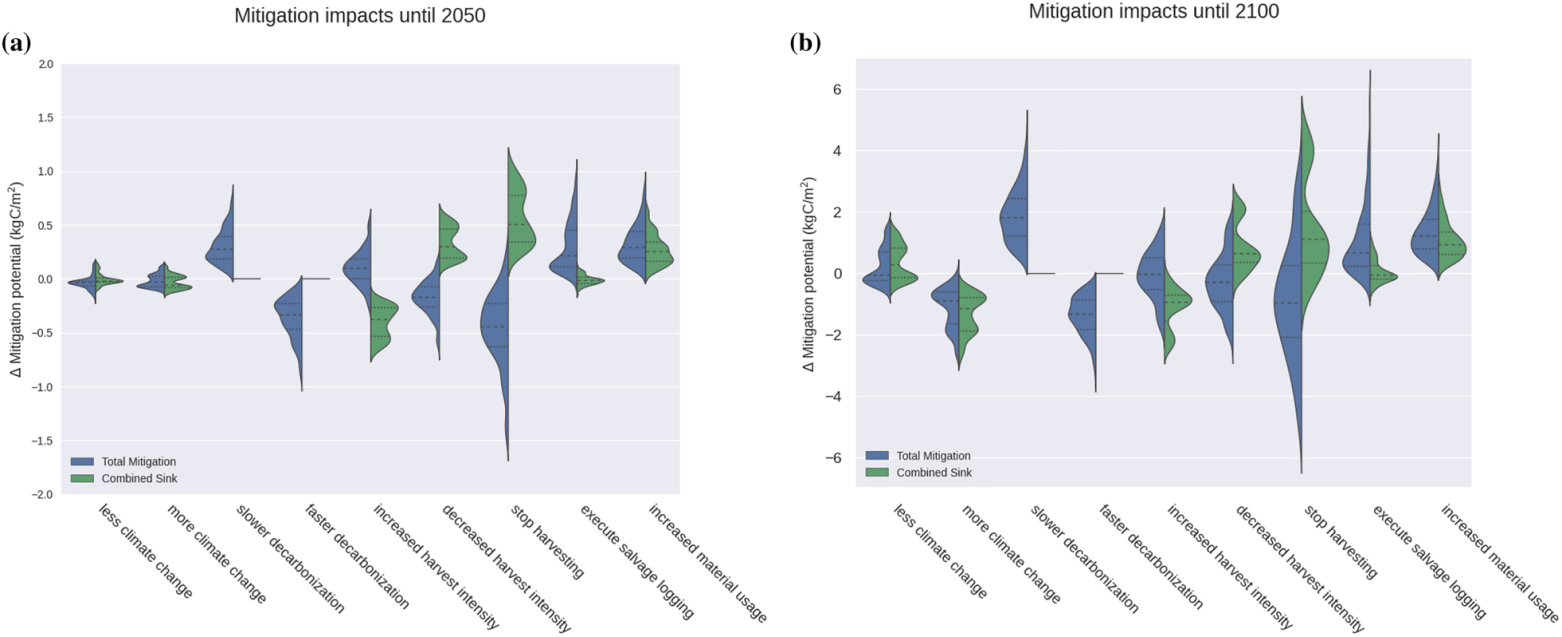
Differences in mitigation potential when changing each considered factor for the time until 2050 (**a)** and 2100 (**b**, note the different scales). Data is based on the assumption of exponential increases in disturbance probability. Each violin plot is created by computing pair-wise differences between simulations with a change in one factor, see section Assessment of the carbon mitigation potential. The split densities show the change in total mitigation potential including substitution effects (blue) and the change in the combined carbon sink only (forest and products). Positive values denote that a higher mitigation potential was simulated compared to its “partner simulation”. In the carbon sink case, this means that it was either a larger carbon sink or a smaller carbon source compared to the “partner simulation”. Note that a negative value does not mean that it was a carbon source. The carbon sink was only smaller than in the “partner simulation”. “More climate change” means subtracting the values of RCP4.5 from the values of RCP8.5, while “less climate change” means subtracting the values of RCP4.5 from the values of RCP2.6. Similarly for decarbonization where the changes to the intermediate 50% decarbonization pace were computed

**Fig. 4:**
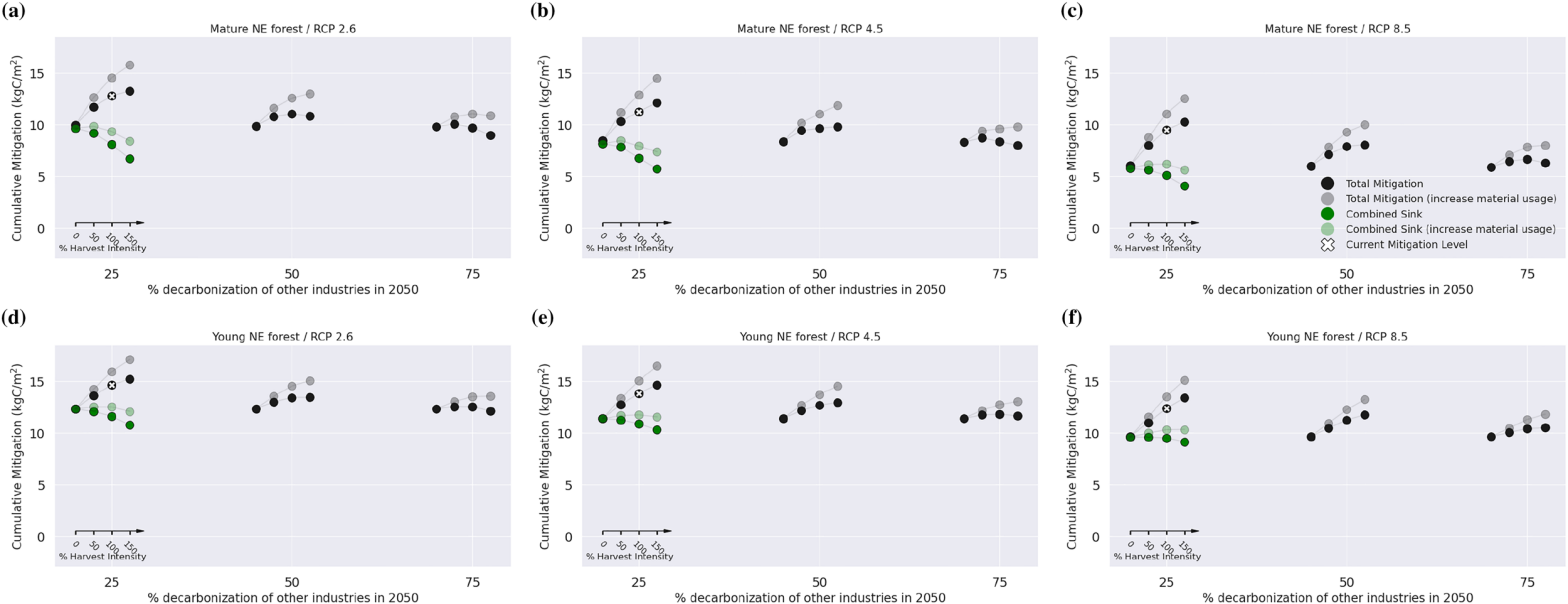
Carbon mitigation potential from 2020 to 2100 for needleleaved evergreen forests. The effects of harvest intensity, wood usage, and decarbonization of substituted materials on the carbon sink and total mitigation potential are displayed for mature forests (**a**–**c**) and young forests (**d**–**f**). The columns show different RCPs. Each group of dots represents one usage scenario and four harvest intensity scenarios (0%, 50%, 100%, and 150%). Black dots represent total mitigation, green dots represent the carbon sink (including products). The darker dots represent current wood usage patterns while the lighter dots refer to simulations with a 50% increase in both material and cascade usage. In low decarbonization scenarios, the substitution effect is so high that increased harvest intensity leads to higher mitigation (black dots). This is not the case when substituted materials become “greener”. The combined carbon sink becomes lower with higher management (including the product sink, as depicted here in green). Shown are the simulations of the results without salvage logging

NE forests had slightly higher mitigation potentials than their BD counterparts, and young forests had a considerably higher mitigation potential than their mature counterparts (Fig. 2). Figure 3 shows a summary of the impacts of all considered factors on the carbon sink and total mitigation potential while Figs. 4 and S8 show these impacts in more detail. In general, the impact on the carbon sink was often different from that on the total mitigation potential. The impact of the factors on the different forest types was rather similar, but in a few cases the effect had a different magnitude, e.g., changes in harvest intensities had a stronger effect on mature BD forests than on mature NE forests (Additional file 1: Fig. S6). Also the time frame mattered: For instance, in the medium term (until 2050) decreasing or stopping harvests generally increased carbon storage but had a negative effect on the total mitigation potential. In the long term (until 2100), however, the total mitigation potential increased for a substantial proportion of simulations for stopping harvests. In general, the spread in mitigation potentials increased with time, due to increasing uncertainty about climate, disturbances, and decarbonization.

#### Forest type

The simulated carbon stocks and sinks were rather similar between NE and BD forests, ranging around $5.5 \,\text {kgC}/\text {m}^2$ for mature forests until 2100 under RCP4.5 (Figs. 2b+Additional file 1: Figure S7). The product sink was much smaller for BD forests ($0.4 \,\text {kgC}/\text {m}^2$ compared to NE’s $1.2 \,\text {kgC}/\text {m}^2$ for mature forests until 2100, Fig. 2), because hardwood is rarely used for long-lived products, unlike softwood. Substitution effects were slightly higher for NE forests, but the contribution of fuel substitution was larger for BD forests. Furthermore, towards the end of the century, disturbances had a pronounced effect on NE forests, leading to lower vegetation carbon stocks. This was, however, counterbalanced by their larger wood product pools (Additional file 1: Fig. S7).

#### Forest age

Forest age also affected the mitigation potential. Young forests in the simulations provided a larger carbon sink (about $9.7\, \text {kgC}/\text {m}^2$ until 2100) than mature ones (about $5.5\, \text {kgC}/\text {m}^2$ until 2100). However, they provided a different product portfolio, leading to a smaller share of long-lived products, particularly in NE forests. Until 2050, the product sink was simulated as 8% of the forest sink for young NE forests compared to 15% for mature ones, while this share was 4% and 5% for both young and mature BD forests, respectively. Substitution effects were also smaller in young forests (Fig. 2).

#### Climate change

The impacts of climate change, including the related exponential increase in disturbance probability based on temperature anomaly, did not fully materialize by 2050, leading to a narrow spread in the changes of total mitigation potentials (Fig. 3). More warming (RCP8.5 instead of RCP4.5) changed the total mitigation potentials by $-0.1$ to $+0.1 \,\text {kgC}/\text {m}^2$, while less warming (RCP2.6 instead of RCP4.5) changed them by $-0.2$ to $+0.1\, \text {kgC}/\text {m}^2$.

In contrast, until 2100, the climate change scenarios exhibited a wider spread in the differences in mitigation potentials: less warming yielded $-0.6$ to $+1.6 \,\text {kgC}/\text {m}^2$ while more warming led to a decrease in mitigation potentials of 0.0 to $-2.6\, \text {kgC}/\text {m}^2$. The interaction between climate change and forest types was significant, with a stronger response in mitigation potential for NE forests compared to BD forests (e.g., compare Additional file 1: Fig. S6 f, h).

#### Disturbances

The assumed temperature-related disturbance scenario had a substantial impact. In the default exponential case, a change from RCP4.5 to RCP8.5 led to a change in total mitigation potential by 2100 of $-1.2 \,\text {kgC}/\text {m}^2$ in the median (Fig. 3). In the linear case, however, the median change until 2100 was only $-0.3 \,\text {kgC}/\text {m}^2$, and in the constant case, $-0.1 \,\text {kgC}/\text {m}^2$ (Additional file 1: Figs. S9, S10). In the constant case, changing from RCP4.5 to RCP2.6 even had strictly negative impacts on the mitigation potential until 2100, indicating positive effects from climate change when no changes in disturbances are assumed.

When focusing on RCP4.5, our results showed a 31% higher carbon sink in mature NE forests by 2100 for simulations without increasing disturbances compared to those with an exponential increase (Additional file 1: Fig. S11). The remaining results remained largely unaffected by the disturbance scenario except for the impacts of salvage logging practices, and minor changes in the effects of decreasing or increasing harvests.

#### Decarbonization

The pace of decarbonization had no effect on the carbon sinks. Consequently, the densities in Fig. 3 are single points, also because we ignored emissions from forestry operations and considered the local decarbonization independent of global emissions. However, it heavily affected the substitution effects: Until 2050, they contributed to 9%-34% of the total mitigation potential for the 100% harvest scenario, depending on the decarbonization pace and the forest type (Fig. 2). Until 2100, faster decarbonization (75% instead of 50% by 2050) decreased the total mitigation potential of the forests by $1.3 \,\text {kgC}/\text {m}^2$ in the median. Similarly, slower decarbonization (25% instead of 50%) by far had the highest positive effect on the mitigation potential of the forests, with $+1.8 \,\text {kgC}/\text {m}^2$ in the median.

Finally, it is notable that by 2100, the absolute impact of slower decarbonization exceeded that of faster decarbonization. This is because, by 2100, the carbon intensity in both the 50% and 75% decarbonization scenario was rather low (3% and 16% of today’s value, respectively), while it remained high in the 25% scenario (47%, see Additional file 1: Fig. S5).

#### Changes in management

Increasing the harvest intensity had strictly negative effects on the combined carbon sink ($-0.2$ to $-0.7 \,\text {kgC}/\text {m}^2$ until 2050 and $-0.0$ to $-2.5 \,\text {kgC}/\text {m}^2$ until 2100, Figs. 3,  4) while decreases in harvest intensity had opposite outcomes. This effect was higher for BD forests than NE forests and for mature compared to young forests (Fig. S6). Changing the harvest intensity heavily affected the importance of the product sink. Higher harvests increased the importance of the product sink, contributing over 30% of the combined sink for mature NE forests in both time frames (Additional file 1: Fig. S12).

The influence of such changes on the total mitigation potential, however, was different. Until 2050, the change in total mitigation potential driven by decreased harvests was mostly negative, ranging from $-0.7$ to $0.2 \,\text {kgC}/\text {m}^2$. Until 2100 it spanned $-2.4$ to $+1.8 \,\text {kgC}/\text {m}^2$. This was driven by substitution effects, dependent on the decarbonization pace. For increased harvests, it was slightly more negative than positive: $-1.9$ to $1.6 \,\text {kgC}/\text {m}^2$ until 2100, with the beneficial cases in the highest carbon-intensity scenario (i.e. 25% decarbonization). But until 2050, there were more instances where increased harvests were beneficial. Regardless of the climate change scenario, benefits of decreased harvests until 2100 almost exclusively occurred in the 75% decarbonization scenario and when there was no increased material usage (Additional file 1: Fig. S13). Note that this result was largely independent from the disturbance assumption Additional file 1: Fig. S14).

Stopping management completely in 2020 increased the combined sink by $0.2 \,\text {kgC}/\text {m}^2$ to $1.0 \,\text {kgC}/\text {m}^2$ until 2050, with BD forests showing a larger resulting carbon sink compared to NE forests (Additional file 1: Fig. S6). Until 2100, the combined sink increased by $0.3 \,\text {kgC}/\text {m}^2$ to $2.8\, \text {kgC}/\text {m}^2$. With stronger climate change and increasing time, this positive impact on the sink decreased, especially for NE forests. In a few cases with NE forests, strong climate change and otherwise high material usage, stopping harvests led to a negative impact on the carbon sink. The impact on the total mitigation potential, however, was almost exclusively negative until 2050 (median $-0.4$ kgC/m2), but spread between $-5.1$ and $3.4 \,\text {kgC}/\text {m}^2$ until 2100.

#### Salvage logging

Salvage logging after disturbances had small effects on the combined carbon sink ($-0.1$ to $0.1 \,\text {kgC}/\text {m}^2$ until 2050, $-0.7$ to $1.1 \,\text {kgC}/\text {m}^2$ until 2100, Fig. 3). The sign of the effects depended on future climatic conditions (temperature and moisture affecting how fast deadwood decays) and usage patterns (depending on whether the residence time of products exceeded that of on-site deadwood). The effects on the total mitigation potential were consistently positive until 2050 and predominantly positive until 2100. Notably, assuming constant disturbance rates decreased the magnitude of the impact. but the generally positive impact remained, regardless of the disturbance scenario (Additional file 1: Fig. S10).

#### Increased material usage and cascading

As expected, an increase in the material usage of wood (i.e., higher shares of long-lived products and more cascade usage) consistently had a positive impact on both carbon sink and total mitigation. The influence of the combination of the two measures on the combined carbon sink was between 0.1 and $0.6 \,\text {kgC}/\text {m}^2$ until 2050 and 0.3 and $2.8 \,\text {kgC}/\text {m}^2$ until 2100. The effect on the total mitigation potential was more pronounced, spanning 0.1 to $0.9\, \text {kgC}/\text {m}^2$ until 2050 and 0.3 and $4.0 \,\text {kgC}/\text {m}^2$ until 2100 (Fig. 3). Thus, increased material usage exerted an important positive effect, regardless of the time frame or the uncertainty from climate and decarbonization scenarios. However, its effect was lower in younger forests compared to mature ones, at least until 2050 (Additional file 1: Fig. S6). After that, the trees in young forests had become similarly large to those of the initially mature forests, making them similarly suitable for long-lived products.

## Discussion

Numerous studies have investigated the carbon mitigation potential of temperate forests. However, these studies often diverge in their findings since they are restricted to a few key assumptions and neglect the corresponding uncertainties. Our study quantifies the impacts and uncertainties associated with the different factors, offering a range of insights for mitigation strategies.

### Effects of external conditions

#### Forest type

Our model simulations indicated similar growth patterns for BD and NE forests. LPJ-GUESS models productivity and growth of trees based on individual characteristics such as their morphology and physiognomy, which affect factors like the amount of absorbed radiation. Also site conditions such as available water and nutrients play a role, which are affected by the composition of tree species at the given site [29]. This simulated similar growth of NE and BD forests might sound counter-intuitive since NE species are known for their rapid growth, but this growth refers to volume, not carbon. BD trees possess a substantially higher wood density than NE trees. For instance, the common NE species of central Europe, spruce and pine, have wood densities of approximately 0.4 to $0.5\,\text {t}\,\text {m}^{-3}$, while the typical BD species, oak and beech, have wood densities of around $0.7\,\text {t}\,\text {m}^{-3}$ [60]. The similar carbon assimilation of these species is manifested in yield tables and observations [61–63].

The difference in wood product portfolios provided by the different forest types is not unique to our study region but common across Europe [64]. Especially NE forests provided a considerable product sink (15% of the forest sink for mature NE forests until 2050) highlighting its importance for mitigation strategies. Consequently, adaptation efforts in Europe towards more BD species will alter the product portfolio and could thus decrease the product sink. One solution to address this are new technologies enabling the usage of BD wood in construction, such as the emerging construction timber products made from European beech [18]. Furthermore, our results indicate that substitution effects are of high importance, regardless of the forest type, because also medium-lived products can exert a large substitution effect [15]. Finally, the higher susceptibility of NE forests to disturbances increases the risk for strategies that decrease or halt harvests, compared to BD forests.

#### Forest age

Younger forests exhibited a greater forest sink but their product sink and substitution effects were smaller (Fig. 2). Age structure also influences resistance against disturbances and maintaining a diverse age structure is proposed as an adaptation measure [65]. In our simulations, we managed forests with a “closer-to-nature” approach, yielding a diverse age structure (Additional file 1: Fig. S2), but future work should consider the likelihood of disturbances in relation to forest structure which we only represented by species-specific disturbance probabilities. Furthermore, different levels of forest productivity should be taken into account [66].

#### Climate change

Climate change can significantly impact mitigation potentials, but this factor is often excluded in mitigation studies [22, 23, 27, 66]. Positive effects on the carbon sink such as $\mathrm {CO_2}$ fertilization and prolonged growing seasons stand in contrast to negative ones such as enhanced drought stress, and increased frequencies of disturbances. Also reductions in nitrogen deposition can play a role [8]. The considered time frame is critical in determining whether positive and adverse effects cancel each other out or not [see also 67]. Our simulations indicated that the differences between RCPs were minor until 2050 (Fig 3) because the disturbance frequencies, $\mathrm {CO_2}$ fertilization, growing season length, etc. remained similar between scenarios.

However, the differences became significant when projecting until 2100. This long-term view remains important because of the generally long time frames in forestry, and because a permanence in carbon storage beyond 2050 is desired (Fig. 3). The effect size also hinged on forest type, with disturbances posing a particular threat to NE forests. In addition, the simulated NE trees were less adapted to warm temperatures, leading to impaired (re-)establishment. Furthermore, a larger carbon stock is at risk in mature compared to young forests. Consequently, the negative impacts of climate change were most pronounced in mature NE forests (Additional file 1: Fig. S6). The fact that BD forests are better adapted to a changing climate is in line with the literature [43, 68, 69]. Therefore, ongoing forest adaptation efforts in Europe towards larger shares of BD species may positively affect mitigation by protecting the in-situ forest carbon sink. However, it is important to note that also broad-leaved trees have started to be impacted by, e.g., droughts [70, 71].

#### Disturbances

We based our results on disturbance frequencies that increased exponentially with temperature (Additional file 1: Fig. S1). Altering this assumption introduced substantial variations in total mitigation potentials (compare Fig. 2 to Additional file 1: Fig. S11). When assuming constant disturbance rates, there were positive effects when transitioning from the RCP2.6 to RCP4.5 scenarios, with a relatively minor difference between RCP4.5 and RCP8.5 (Additional file 1: Fig. S10). Even until 2100, the difference in RCPs was much smaller compared to other factors. With linear increases in disturbance frequencies, slightly negative impacts on the mitigation potential emerged going from RCP4.5 to RCP8.5, albeit dampened by positive effects from increased temperatures, CO_2_ fertilization, and prolonged growing seasons (Fig. 1). Scenarios with an exponential increase in disturbance probabilities exhibited substantial negative effects when transitioning to higher RCPs due to disturbances, overshadowing other climate change effects.

It is important to note that LPJ-GUESS employs simplistic models of water stress. Generally, dynamic vegetation models exhibit varied responses to drought and heat [72] and new hydraulic models are necessary for a more comprehensive understanding [73]. Moreover, the anticipated increase in disturbances is a critical aspect that is often overlooked in models, highlighting the significance of accounting for this aspect, as emphasized by our results. Nevertheless, although the magnitude of impacts was affected by the assumption on the disturbance scenario, the qualitative conclusions remained largely unaffected.

#### Decarbonization

The potential impact of anticipated decarbonization is often overlooked in mitigation studies. Wood offers climate benefits when substituted for various materials, including concrete, steel, glass, plastics, and aluminum [15]. Plans to decarbonize these industries are already in motion, driven by enhancements in efficiency, recycling, and product lifetimes, and the promotion of state-of-the-art technologies and innovations [13, 14, 16]. The pace of this decarbonization, however, remains uncertain. Currently implemented EU policies are projected to reach a 25% reduction in gross emissions by 2050, whereas the target is a reduction of about 80% [Additional file 1: Fig. S5, 44].

In this study, the decarbonization pace played a pivotal role, driving differences between the combined carbon sink and total mitigation potential, occasionally resulting in contrary outcomes between the two. As decarbonization accelerates, substitution effects become less significant (Fig. 2). But even in the most ambitious, net-zero-compliant scenario, decarbonization will take time. Within the next decades, wood usage thus remains an important lever for carbon mitigation. Decreased harvests and wood usage would lead to negative substitution effects, i.e., higher emissions. After 2050, the importance of wood products for mitigation will diminish in a fast-decarbonization world, offering the opportunity to focus on other ecosystem services. Conversely, with slow decarbonization, the pressure on forests to provide mitigation will persist beyond 2050. This is evident in the substantial differences in total mitigation impact by 2100 between slower and faster decarbonization. The 25% decarbonization pace is close to projections for current policies, emphasizing that a fast speed-up of decarbonization would remove the pressure from forests to provide mitigation via substitution effects.

Apart from its pace, also the general concept of substitution effects bears uncertainties [56, 74, 75]. Three main issues are additionality (would the wood product have been created anyway?), leakage (was the substituted material not simply used elsewhere?), and replaceability (what type of fuel was replaced by wood?). Here, we neglected these aspects and examined the *theoretical* maximal substitution potential, offering insight into the overall importance of substitution. For instance, if wood production ceased in the EU, an additional 410 Mt $\text {CO}_{{2e}}$ could be emitted annually through alternative products, equivalent to 15% of the EU’s 2022 emissions [4, 7]. Concrete applications of substitution effects require comparing a scenario against a baseline to grasp the true mitigation impact.

### Changes in management

Decreasing harvest intensity enhanced the forest carbon sink. This effect was smaller in NE forests because the increased growing stock led to higher losses from disturbances, particularly until 2100. The effects on the total mitigation potential depended on the time frame. They were clearly negative until 2050, but diverging until 2100 (Fig. 3). In the slow decarbonization scenario (25% decarbonization by 2050), reduced substitution effects more than outweighed the enhanced sink (Figs. 4+S8). Conversely, with faster decarbonization, the increased carbon sink outweighed the decreased substitution effects in most cases. The almost consistently decreased mitigation potentials due to decreased harvests until 2050 indicate that decreasing harvest intensities in sustainably managed forests might be counterproductive from a mitigation point of view.

Halting forest management yielded similar outcomes. While it would have numerous benefits for biodiversity and ecosystem services, doing so in sustainably managed mature forests might not provide anticipated mitigation benefits. Forest growth is eventually reduced, leading to a loss in sink strength. Simultaneously, climate change and associated disturbances threaten forests with high growing stocks.

Studies suggesting that decreased harvest levels or prolonged rotations are beneficial for mitigation only seemingly contradict our findings. Their regional DFs for material substitution (0.45$-$0.6 tC/tC, [22, 27, 76]) are considerably lower than ours (1.5 tC/tC). One important reason for this discrepancy is landfilling: in the meta-analysis by Sathre and O’Connor [77], average material DFs are 2.1 tC/tC and 1.1 tC/tC without and with landfilling (partially even including methane recovery), respectively. The aforementioned studies are thus more comparable to our results with 75% decarbonization where also in our simulations decreased harvests had a total mitigation benefit in most cases (Figs. 4 and Additional file 1: Fig. S8). Consequently, it is critical that DFs are assessed regionally and thoroughly, including end-of-life treatment, because their magnitude heavily impacts the mitigation impact of different management intensities.

Many mitigation studies were conducted in Northern Europe, where the forest carbon sink has been particularly strong due to the prevalence of young stands [23, 27]. In our simulations, the contribution of the carbon sink to total mitigation was also higher in young stands (Fig. 2). Furthermore, in Scandinavia, larger fractions of wood are used for pulp and energy than in Bavaria, even for conifers [27]. Also, unlike here, negative impacts of climatic change were either not considered in the cited studies, or only simplified [78, 79], potentially leading to an overestimation of the forest sink’s contribution to mitigation. In our simulations, for instance, removing the temperature-dependent increase in disturbance frequencies led to a 15–30% higher carbon sink in NE forests until 2100 (Additional file 1: Fig. S11).

Increased harvests in our simulations had mostly positive effects on mitigation until 2050, aligning with findings in studies employing high DFs [80]. However, considering the likely decrease in substitution effects, this positive effect only persisted until 2100 for slow decarbonization paces, where high substitution effects outweighed the decreased forest sink. For faster decarbonization, increasing harvests had strictly negative effects, aligning with other studies, e.g. [81] who advocated against increasing harvests with DFs less than 1.1 tC/tC. Also Skytt et al. [66], using multiple DFs (e.g., 0.7–2.8 tC/tC for sawn wood), arrived at a similar conclusion: with strongly decreasing substitution effects, less harvesting is beneficial.

The harvest routines implemented in this study are very simplified, but different management regimes and replanting schemes could potentially provide co-benefits for the carbon sink and harvest volumes. These were beyond the scope of this study but need to be assessed in future work. Furthermore, we did not consider clearcuts because they are not allowed in Germany on a large scale and would not be compatible with other demands on forests. LPJ-GUESS also does not model the important impacts on micro-climate, and thus cannot simulate the impaired reestablishment after clearcut [e.g., 82].

#### Salvage logging

Salvage logging is a controversial [53, 83–85], yet common and in some European regions mandatory practice [86, 87]. Here, we quantified its direct impacts on the carbon balance, excluding considerations related to preventing subsequent disturbances, micro-climate effects, or habitat provisioning.

Whether salvage logging is beneficial for the carbon balance depends chiefly on wood usage and the climate scenarios that determines how fast the wood decays if left in the forest. Our simulations generally indicated a modest and ambiguous impact of salvage logging on the carbon sink, in line with studies showing similar decay times of deadwood and wood product portfolios [11, 88–91]. In our simulated BD forests, however, salvaging had mostly negative impacts on the carbon sink, because BD wood is predominantly burned (Fig. 3).

In terms of total mitigation potential, salvaging was mostly beneficial. Its impacts were particularly high in NE forests under RCP8.5, driven by high disturbance frequencies, a faster decay of deadwood, and a product portfolio with long lifespans.

While we considered that not all wood undergoes salvage logging after disturbances [53], we did not adjust wood usage patterns. It is difficult to get estimates on the use of salvaged wood, but statistics from Germany indicate no substantial change to default patterns (Additional file 1: Fig. S3). Depending on the preceding disturbance, salvaged wood may be as effectively utilized as fresh wood [92]. However, post-disturbance logging disrupts wood markets, usually increasing exports [93]. This poses another complexity in estimating mitigation benefits, as transport emissions rise, and displacement effects become more challenging to estimate.

### Wood products

The advantages of increased material usage align with other studies [9, 20–23]. This benefit was relevant across all decarbonization scenarios because of the enhanced product sink (Fig. 4). However, the earlier measures are implemented to facilitate increased material usage, the greater their impact because of substitution effects. This key role of material substitution, especially in the coming decades, was also highlighted by Nabuurs et al. [80].

This importance of wood products seems to contradict recent studies [94] who suggested a limited significance of the global product sink. However, their and other studies, e.g., [66], used IPCC’s global estimate of 35 years as the maximum half-live of long-lived products. Here, we used much higher residence times from regional analyses. Even more importantly, we used decay rates based on Gamma functions. These, unlike the usual exponential functions, account for a lag in decay after product creation (Additional file 1: Fig. S4). Even with these high estimates, our simulated product sink is in the order of 10% of the forest sink, aligning with other estimates [6, 7, 9, 19]. This underlines that wood products in regions with sustainable harvests and high material usage are important for the mitigation potential. This significance is further magnified when substitution effects are considered. Nevertheless, it is necessary to mention that we neglected local changes in the wood industry (e.g., scaling effects) and trade impacts that are relevant in the study region [5]. Both affect product portfolios and substitution effects, but their detailed assessment is beyond the scope of this study.

#### Feasibility of other harvest intensities and wood usage patterns

Unlike changes in wood usage, modifications of harvest intensity affect various ecosystem services such as habitat provision, recreation, or local climate regulation through biophysical effects. Changes in the latter could even offset positive impacts on atmospheric $\mathrm {CO_2}$ concentrations [95–99]. It is crucial that forests are not only considered for their mitigation potential, but also for their manifold ecosystem services [51, 100]. We agree with [24], proposing that forest management strategies should consider a desired sink strength and then estimate how much wood can be safely removed from the forest.

However, wood demand has been rising and is projected to continue increasing [101]. A reduced timber supply could lead to negative substitution effects with highly carbon-intensive products replacing wood. While some additional wood could be directly used with present-day construction methods, some additional usage likely depends on technological advances [17, 18, 75]. These necessitate new building codes, worker training, and public acceptance [74]. However, we assumed a linear increase in usage, allowing these changes to occur at a similar pace as historically observed in the study region [19].

Increased harvest intensities lead to smaller trees and a different product portfolio. Our assumptions about increased wood use thus also rely on the belief that parts of the stem currently allocated for short-lived products can serve longer-term purposes. Cross-laminated timber, for instance, can be derived from low-value wood [102]. Moreover, while a significant portion of needle-leaved trees already serves construction purposes in Bavaria, achieving a 50% increase (from 37% to 55.5%) is challenging. For broad-leaved trees, however, there is greater potential, given their currently low utilization (6%).

#### Impact on fuel provision and short-lived products

Promoting long-lived products decreases the share of short-lived products and fuel wood. Heated debates revolve around the climate effects of wood fuels, which many governments currently consider carbon-neutral, assuming eventual forest regrowth [103, 104]. This assumption is questionable given increasing disturbances and eroding forest resilience. Additionally, wood fuels emit more $\mathrm {CO_2}$ per unit of energy than fossil fuels (indicated by a $\text {DF} < 1$) and it is unclear which energy sources wood fuels replace. This makes the carbon impact of wood fuels debatable [103–108].

In the EU, about one-fourth of all roundwood harvests currently serve as fuel wood [109], but all combined direct and indirect wood supplies (i.e., including recycled wood) contribute only about 6% to total gross final energy consumption [110]. Since wind and solar can generate significantly higher amounts of energy per area than bioenergy [111, 112], it should be possible to compensate for a gradual reduction in wood fuel provision via alternative energy sources. Nevertheless, energy security has recently become a major societal concern again in Europe due to the Russian war in Ukraine. This increases the importance of locally available fuels like wood. There is also a growing global demand for pulp and paper products, largely driven by increased packaging and sanitary paper needs [113]. These developments directly conflict with the increased provision of long-lived products assumed in this study and may require reduced usage and enhanced recycling efforts.

## Conclusion

Optimizing forestry for mitigation, while simultaneously considering other ecosystem services, is one of many important strategies to mitigate climate change and complements the urgent need to reduce emissions. However, the multitude of factors determining the mitigation potential and their interactions have often been excluded in previous studies, making it difficult to draw general conclusions. In this study, through factorial modeling experiments for Bavaria as an example of the central European domain, we assessed a wide range of such factors: forest age, forest type, climate change and nitrogen deposition, disturbances, harvest intensity, salvage logging, wood usage, and the carbon intensity of other industries. Our approach allows us to suggest eight recommendations for forest-based mitigation assessments.

1. Climate change impacts (especially disturbances) and decarbonization are among the most important yet uncertain factors influencing mitigation and must not be neglected. Our analysis indicates that until 2050 these uncertainties are narrow enough to confidently develop mitigation projections. Looking beyond 2050 (which is necessary due to the long time spans in forestry), we suggest utilizing robust methods and risk diversification to account for the large uncertainties.
2. Increasing climate change enhances pressure on forests, especially through disturbances. In that regard, global climate change mitigation offers co-benefits for forest health and local forest-based mitigation.
3. The substantial differences in mitigation potentials arising from assumptions about changes in disturbance frequencies highlight the necessity for further model improvements.
4. Mitigation strategies need to be tailored to local forest conditions. Forest age and type heavily influence mitigation potentials, e.g. through different growth dynamics or product portfolios. Adaptation efforts towards more BD species – crucial to foster resistance to climate change – should be accompanied by the promotion of technologies to use more hardwood for long-lived products, thereby maintaining the product sink and maximizing substitution effects.
5. Substitution effects and the magnitude of DFs are crucial factors determining the mitigation potential. A thorough quantification of DFs, including end-of-life management of wood products, should be a key research priority.
6. Our simulations suggest that decreasing or stopping harvests reduces the mitigation potential in the considered region, especially until 2050. The product sink and substitution effects are still high and dismissing them would outweigh the increased forest carbon sink. In the long-term, this may change, but increased growing stocks then are at higher risk to be affected by disturbances. Modest increases in harvest intensity could provide mitigation benefits until 2050 depending on forest characteristics and decarbonization pace, but likely at the cost of other ecosystem services.
7. Increased material usage has a clear climate benefit, regardless of the scenario. However, the trade of wood products and other economic aspects affecting the mitigation potential need to be addressed in future studies.
8. Delaying decarbonization puts long-term pressure on forests to provide mitigation and puts forest health at risk. Any speed-up in decarbonization will thus greatly lift the pressure off forests and allow forest management to focus on other ecosystem services.

Our study provides a foundation for evaluating the carbon mitigation potentials of managed central European forests by quantifying key factors and uncertainties. These need to be taken into account when developing forest-based mitigation strategies, all the while keeping in mind the broader value of forests in providing numerous ecosystem services.

### Supplementary Information

**Additional file 1. Supplementary Figures.**

## Data Availability

All code and data to reproduce the results and figures are available at https://github.com/k-gregor/carbon-mitigation

## Abbreviations

BD: Broad-leaved deciduous
DBH: Diameter at breast height
DF: Displacement factor
NE: Needle-leaved evergreen
RCP: Representative concentration pathway
N: Nitrogen
