# Supplementary material for: Quantifying the impact of key factors on the carbon mitigation potential of managed temperate forests
Source: Carbon Balance Manag. 2024 Mar 2;19:10. doi: 10.1186/s13021-023-00247-9 (PMC11342660; doi:10.1186/s13021-023-00247-9)
Supplement: Supplementary file 1 — Additional file 1. Supplementary Figures. [file 13021_2023_247_MOESM1_ESM.pdf]

Supplementary Material: Quantifying the impact of key factors on the carbon mitigation potential of managed temperate forests

Konstantin Gregor[1,*], Andreas Krause[1], Christopher P O Reyer[2], Thomas Knoke[1], Benjamin F Meyer[1], Susanne Suvanto[3,4,5], and Anja Rammig[1]

[1]TUM School of Life Sciences, Technical University of Munich, Freising, Germany

[2]Potsdam Institute for Climate Impact Research, Member of the Leibniz Association, Potsdam, Germany

[3]Natural Resources Institute Finland (Luke), Helsinki, Finland

[4]School of Geography, Earth and Environmental Sciences, University of Birmingham, United Kingdom

[5]Birmingham Institute of Forest Research, University of Birmingham, United Kingdom

[*]Corresponding author, konstantin.gregor@tum.de

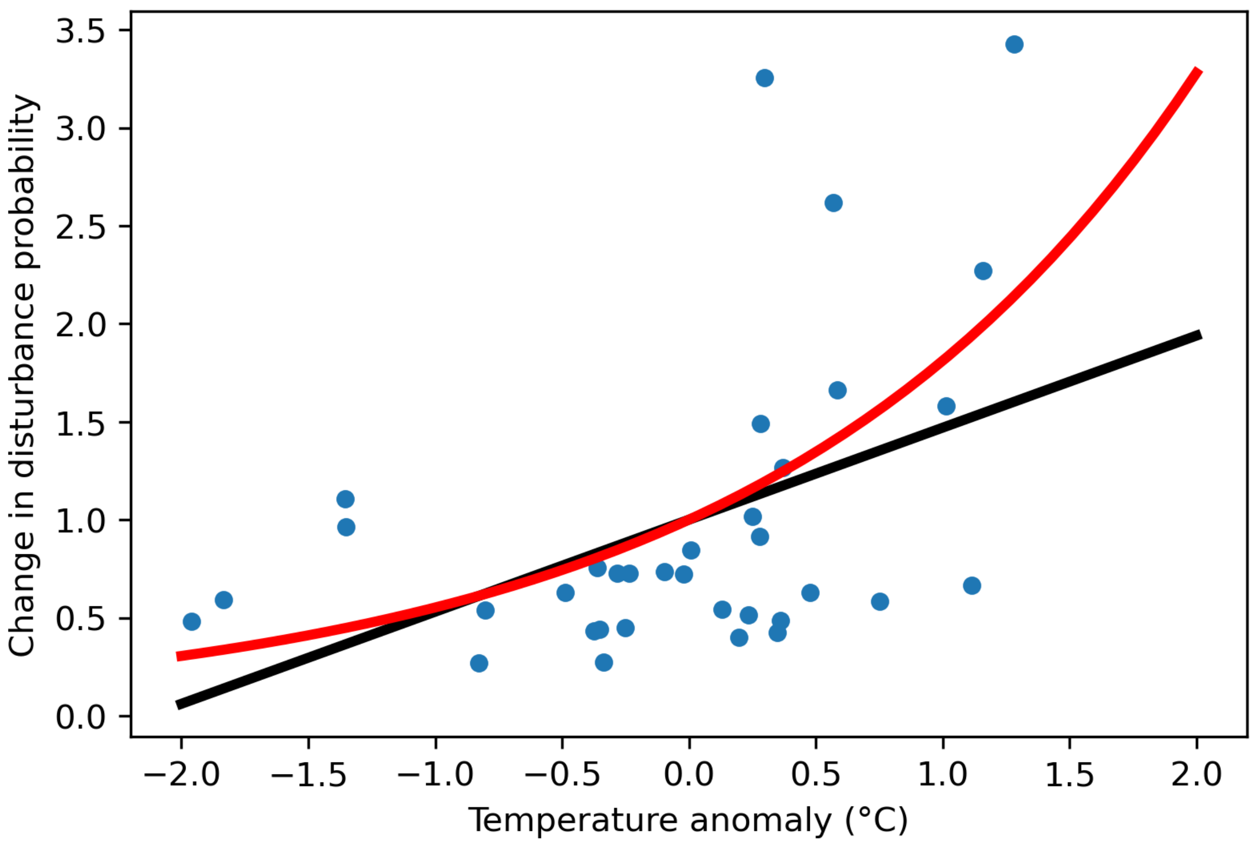

Figure S1: Fitted exponential (red) and linear (black) relationship between temperature and probability of a disturbance based on observed data of disturbances (blue) in Germany from Senf and Seidl (2021). The mean surface temperature of a grid cell of 2001-2014 was used as the baseline temperature as this was the time frame from which the disturbance return times of Pugh et al. (2019) were observed.

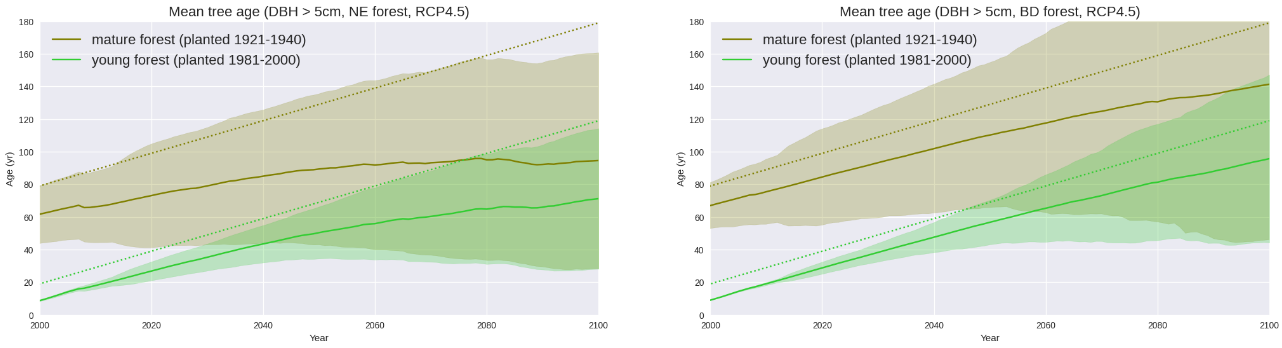

Figure S2: Example of the age structure of the forests for the 100% management scenario, and RCP4.5 with exponential increase in disturbance frequencies. The forests are planted and consecutively thinned, leaving room for new establishments. Depicted is the mean age with bands of one standard deviation. The dotted lines depict the maximum tree age inside the forest.

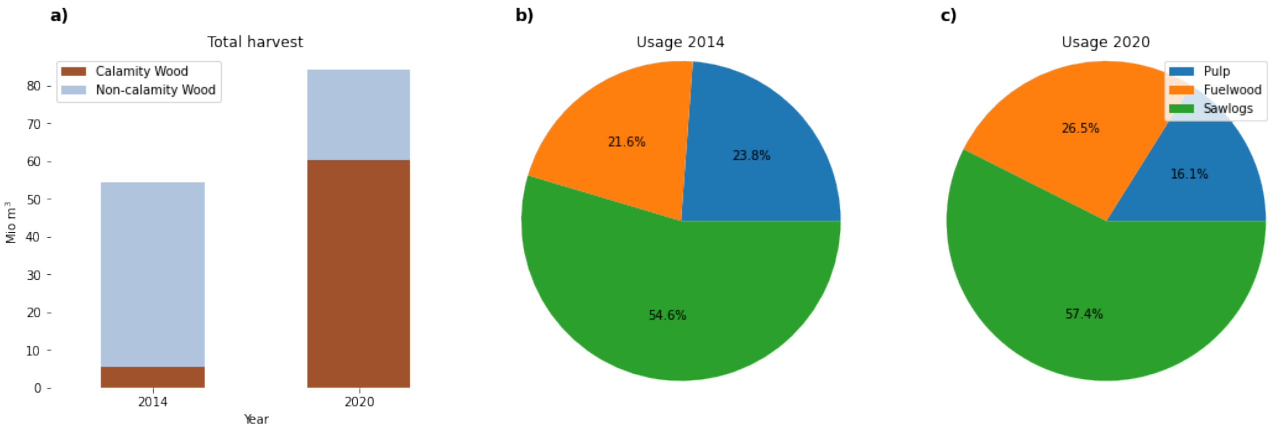

Figure S3: a) In Germany in 2020, about 72% of total wood harvests came from salvage logging, whereas this fraction was only 10% in 2014 (BMEL, 2021; Destatis, 2022). b+c) The resulting wood usage portfolio was not drastically different between the two years.

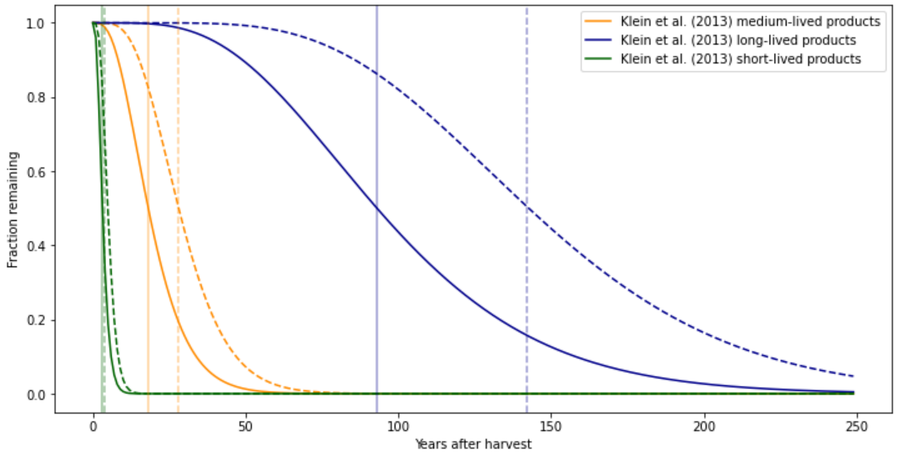

Figure S4: Decay functions for the different product pools. Vertical lines show the median residence time, dashed lines are for the simulation with 50% increased residence time, representing increased cascade usage.

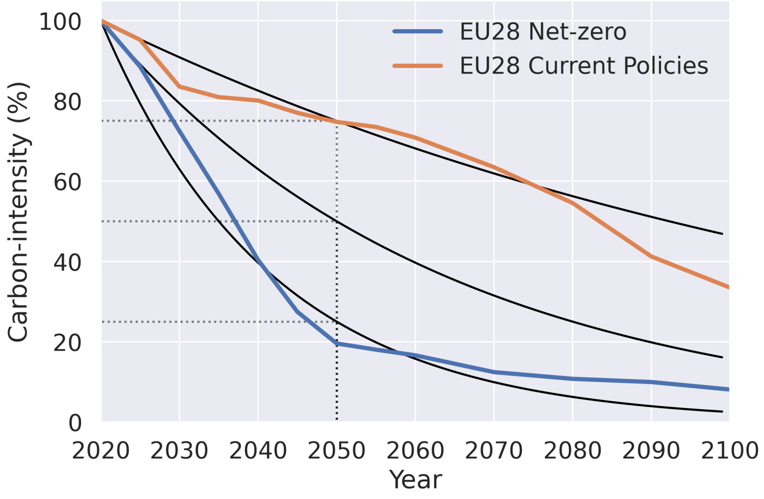

Figure S5: The three considered decarbonization scenarios. They reach 25%, 50%, and 75% decarbonization, respectively. The 25% and 75% scenarios are fitted to closely match the carbon-intensity of the EU with current policies and net-zero policies, respectively, based on data of Schreyer et al. (2020).

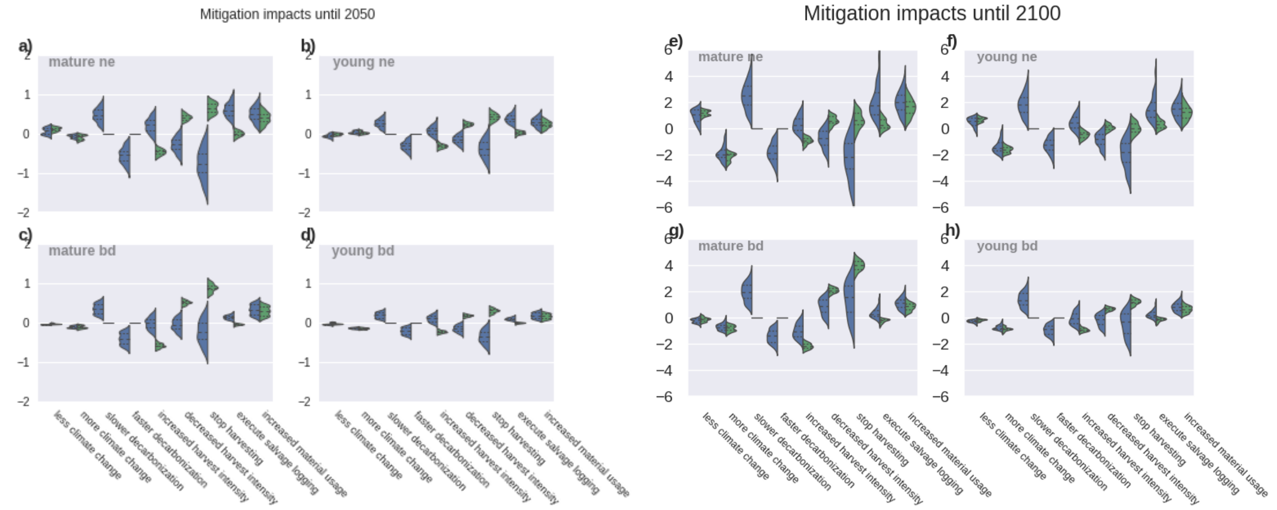

Figure S6: Same as figure 3 but split up into the forest types. Note the different scales for 2050 (a-d) and 2100 (e-h).

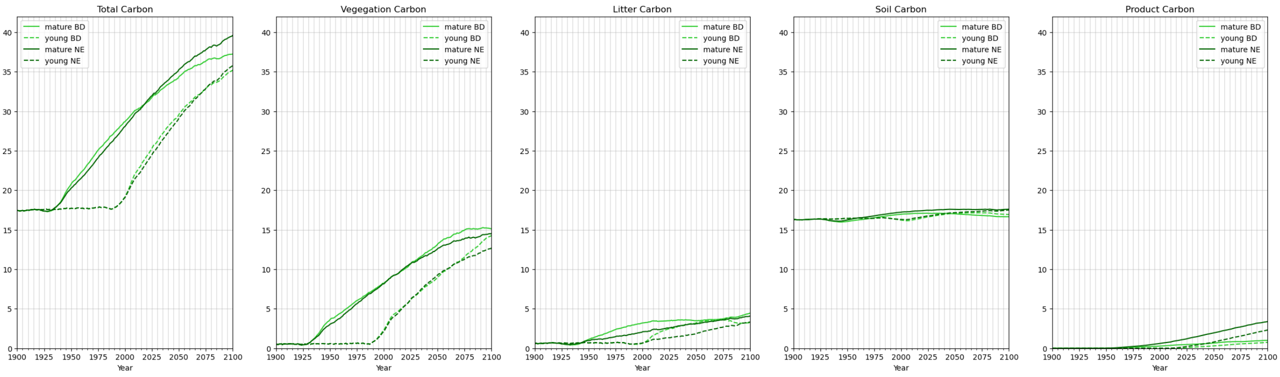

Figure S7: The different carbon pools for young and mature NE and BD forests under RCP4.5 and 100% management.

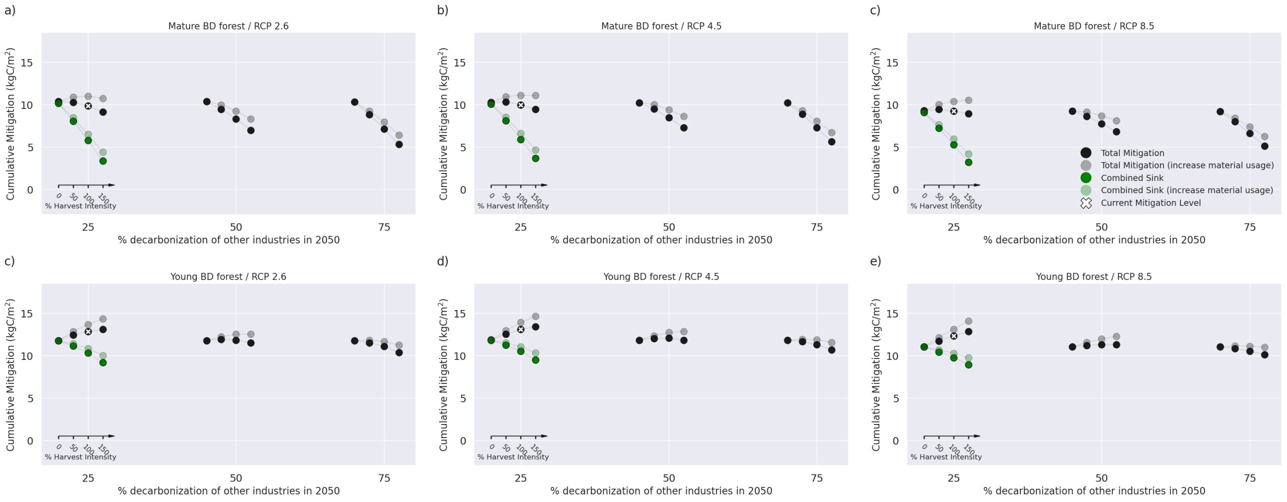

Figure S8: Same as Figure 4, but for BD forests.

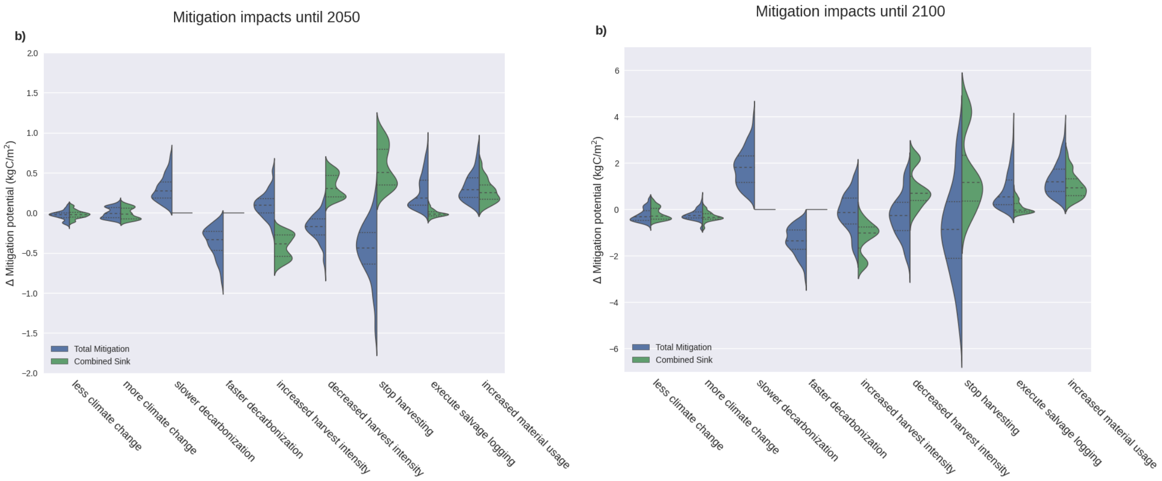

Figure S9: Same as Figure 3 but with a linear increase in disturbance probability based on temperature anomaly.

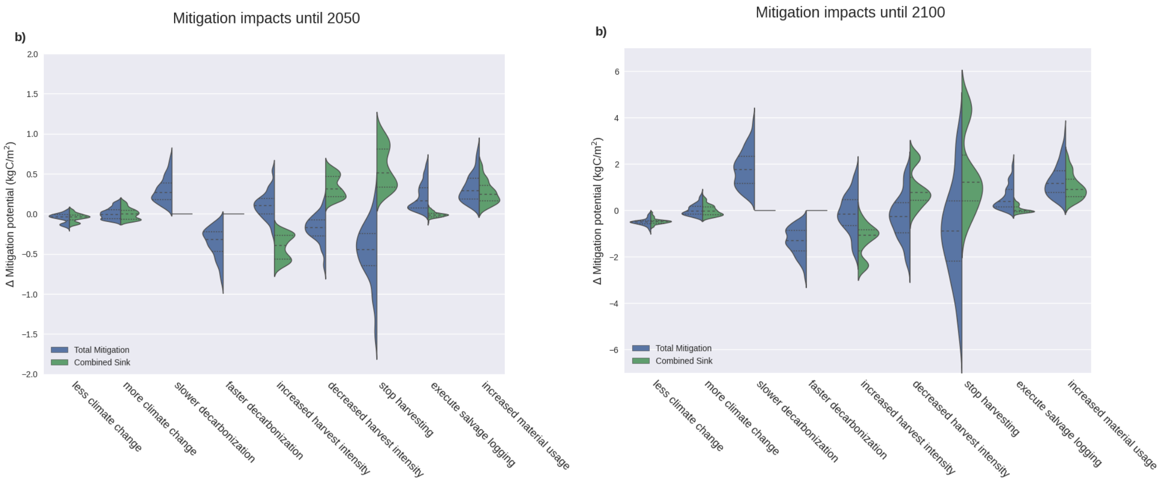

Figure S10: Same as Figure 3 but with constant disturbance rates

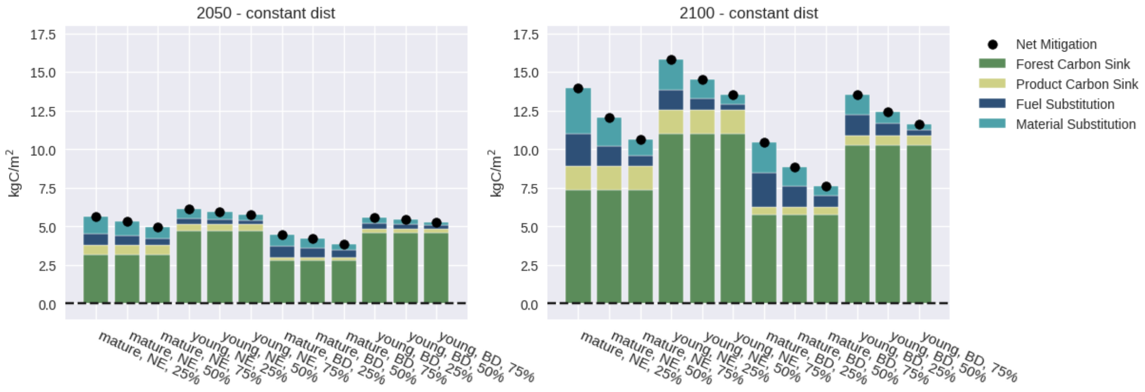

Figure S11: Same as Fig. 2 but without temperature-dependent increases in disturbance frequencies.

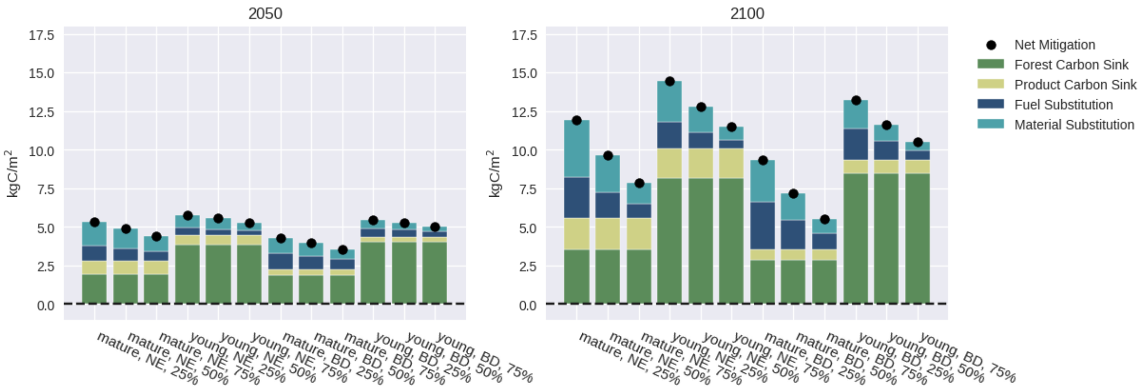

Figure S12: Same as Fig. 2 but with 150% harvest intensity.

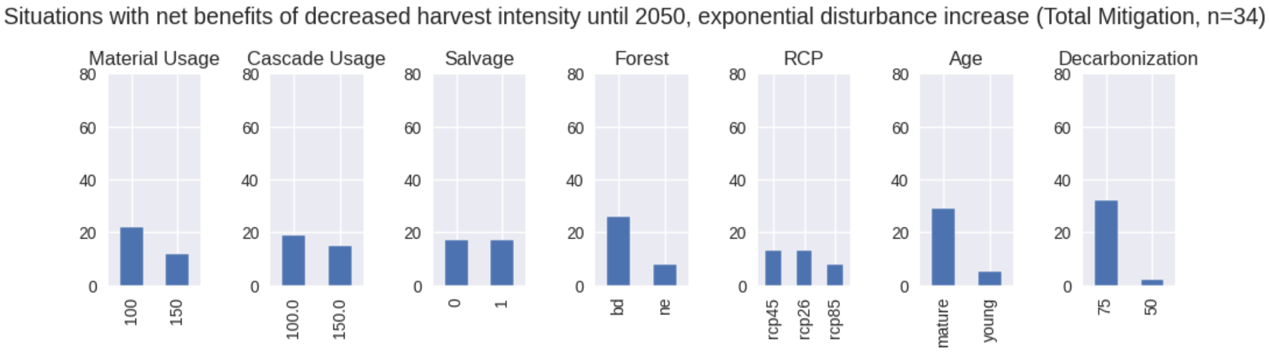

Figure S13: Situations where decreasing harvesting was beneficial for the carbon mitigation until 2050.

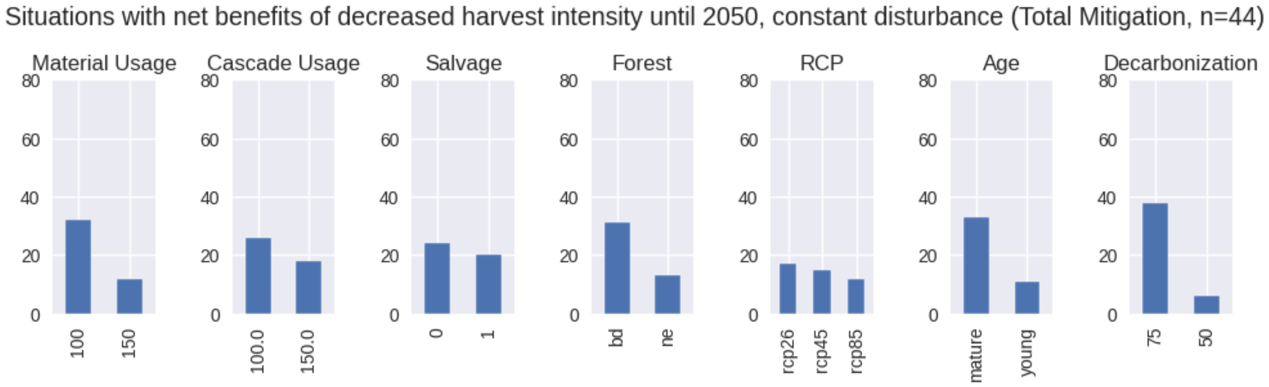

Figure S14: Situations where decreasing harvesting was beneficial for the carbon mitigation until 2050, assuming constant disturbance rates.

References

BMEL. (2021). *Holzmarktbericht 2020* (tech. rep.). Bundesministerium für Ernährung und Landwirtschaft (BMEL). https://www.bmel-statistik.de/fileadmin/daten/FHB-0120005-2020.pdf

Destatis. (2022). Durch Schäden bedingter Holzeinschlag. Retrieved November 9, 2022, from https://www.destatis.de/DE/Im-Fokus/Klima/_Grafik/_Interaktiv/schadholzeinschlag.html

Pugh, T. A. M., Arneth, A., Kautz, M., Poulter, B., & Smith, B. (2019). Important role of forest disturbances in the global biomass turnover and carbon sinks. *Nature Geoscience*, *12*(9), 730–735. https://doi.org/10.1038/s41561-019-0427-2

Schreyer, F., Luderer, G., Rodrigues, R., Pietzcker, R. C., Baumstark, L., Sugiyama, M., Brecha, R. J., & Ueckerdt, F. (2020). Common but differentiated leadership: strategies and challenges for carbon neutrality by 2050 across industrialized economies. *Environmental Research Letters*, *15*(11), 114016. https://doi.org/10.1088/1748-9326/abb852

Senf, C., & Seidl, R. (2021). Mapping the forest disturbance regimes of Europe. *Nature Sustainability*, *4*(1), 63–70. https://doi.org/10.1038/s41893-020-00609-y
